# Perceived Discrimination in Healthcare Settings is Associated with Medication Side Effects and Adherence: A Cross-Sectional Survey Representing the Four Largest Ethnic Groups in the UK

**DOI:** 10.1007/s40615-025-02403-y

**Published:** 2025-03-26

**Authors:** Rebecca K. Webster, Aarti Iyer

**Affiliations:** School of Psychology, University of Sheffield, Sheffield, UK

**Keywords:** Perceived discrimination, Side effects, Adherence, BAME

## Abstract

Many medication side effects are the result of negative patient expectations rather than the pharmacological action of the drugs, thus triggering a “nocebo effect.” Negative expectations can be generated by contextual factors that contribute to the perceived quality of care. Research shows that perceived discrimination in healthcare settings among Black, Asian, and other Minority Ethnic (BAME) groups is associated with lower perceived quality of care. Yet, no work has considered whether perceived discrimination contributes to experiences of nocebo effects among BAME groups, leading to potentially poorer medication adherence and health outcomes. We conducted an online cross-sectional survey of 801 participants representing the four largest UK racial/ethnic groups (Asian, Black, Mixed, and White) who had been prescribed a new medication in a general practitioner (GP) consultation in the previous 6 months. Participants’ perceived discrimination during their GP consultation was significantly and positively associated with their side-effect experience, and significantly and negatively associated with their adherence to the prescribed medication, with side-effect expectations significantly mediating these relationships. There was a significant interaction between ethnicity and perceived discrimination: participants who identified as mixed race were less likely to report side effects than White participants who experienced the same level of discrimination. Future longitudinal studies are needed to confirm the identified causal relationships and to explore the types of perceived discrimination BAME individuals are experiencing in health care to help develop interventions to reduce nocebo effects.

**Supplementary Information:**

The online version contains supplementary material available at 10.1007/s40615-025-02403-y.

## Introduction

Between 38 and 100% of side effects reported to medications are not related to their pharmacological actions [1]. Instead, many “side effects” consist of nonspecific symptoms that patients *believe* are caused by the medication, but arise through a psychological phenomenon known as the “nocebo effect” where noxious symptoms are largely generated through negative expectations [2, 3]. These negative expectations often consist of concerns about the safety of the medication and its potential side effects [4]. Negative expectations can be caused by contextual factors within healthcare settings such as the doctor-patient relationship, and the doctor’s empathy, contributing to the perceived quality of care [5, 6]. Nocebo effects are important to understand and prevent because they can lead to lower medication adherence and thus poorer health outcomes [7].

To date, research on the nocebo effect has investigated patient experiences in general, without systematically considering the fact that not all patients have the same experiences within the healthcare system. Research has documented racial/ethnic group disparities in the quality of health care received by patients, which contribute to negative health outcomes among Black and Minority Ethnic (BAME) communities [8–10]. These disparities in health outcomes are especially pronounced during public health crises: research indicates that BAME groups are at increased risk of death from COVID-19, which cannot be attributed solely to economic deprivation or geographic region [11].

Evidence indicates that racial/ethnic discrimination at least partly underlies disparities in healthcare quality among BAME groups [12]. However, no research has examined how these experiences of discrimination contribute to BAME individuals’ experiences of the nocebo effect, which in turn may account for lower healthcare outcomes among BAME patients [13]. For example, negative interactions with healthcare providers due to discrimination could foster negative expectations that contribute to the experience of side effects from prescribed treatments; these side effects in turn may subsequently affect adherence decisions and therefore health outcomes. The present study addresses this gap.

Our focus on the structural conditions (i.e., racial/ethnic discrimination) that contribute to health outcomes is grounded in the Social Determinants of Health framework, a theoretical model connecting people’s health to the economic, social, and political conditions in which they live [14]. Research demonstrates that inequalities in physical and mental health are in part determined by the unequal distribution of resources that determine health, such as education, housing, and employment. This project will add to this conceptual framework by investigating a novel psychological process (the nocebo effect) that can underpin the relationship between environmental conditions (perceived discrimination) and health outcomes.

BAME communities are generally underrepresented in health research [15], and only a handful of studies have examined the association between discrimination in healthcare and health outcomes. These studies are limited for at least two reasons. First, they use single-item measures capturing only a simplistic assessment of discrimination (often only in relation to race), and thus may miss the broader set of processes and discrimination at play. Second, these studies primarily focus on African American samples within the USA, thus neglecting other minority racial/ethnic groups [12, 15]. Investigating the extent to which perceived discrimination in healthcare settings contributes to the nocebo effect through increased experience of side effects, presents a novel relationship that could elucidate the link between racial/ethnic disparities in health outcomes.

### Research Objectives

We aimed to recruit 800 participants from each of the four largest UK racial/ethnic groups (Asian, Black, Mixed, and White ethnic groups) so that each group made up 25% of the sample. In addition, participants had to have been prescribed medication by their GP in the previous 6 months so they could retrospectively reflect on their experience at the GP clinic and their experience with their subsequently prescribed medication to address the following research objectives: (1) to investigate the associations between perceived discrimination in healthcare settings, experiences of medication side effects, and medication adherence; (2) to assess whether the relationships between perceived discrimination, medication side effects, and adherence vary across different racial/ethnic groups; (3) to examine potential mediators between perceived discrimination and nocebo effects, including relationship with their doctor, perceived doctor empathy and quality of the healthcare service received, and side-effect expectations.

## Methods

### Design and Participants

Participants of at least 18 years of age were invited to take part in an online cross-sectional survey if they were a resident in the UK, and they had been prescribed medication by their GP in the previous 6 months. We used two market research companies to recruit a sufficiently large sample of racial/ethnic groups who met these inclusion criteria. To ensure adequate power for the interaction analyses (as detailed below), quotas of 25% (200) were used for each of the four largest ethnic groups represented in the UK, as per the 2011 Census [16]: *Asian* (Indian/Pakistani/Bangladeshi/Chinese/other Asian background), *Black* (African/Caribbean/other Black background), *Mixed/multiple ethnic groups* (White and Black Caribbean/White and Black African/White and Asian/other Mixed or Multiple ethnic background), and *White* (English/Welsh/Scottish/Northern Irish/British/Irish background).

### Sample Size

Relationships between perceived quality of care and nocebo effects tend to show small effects [6]. A total sample size of 528 was calculated to have enough power to detect a potentially small but significant effect of perceived discrimination on a side-effect experience using regression analyses (*f*^2^ 0.02, *α* = 0.05, power = 90%). We aimed to recruit a total sample of 800 participants (200 from each racial/ethnic group) to meet this minimum requirement and increase the power needed to test for mediation and moderation effects.

### Procedure

Potential participants were notified via the market research companies about the study and received a link to the survey to take part. For one market research company, the survey was sent to those already identified as eligible. For the second, we set up two surveys, a short pre-screening survey and the main survey, and only invited those deemed eligible from the pre-screening survey to complete the main survey. The beginning of the survey contained the information sheet and after providing informed consent, participants completed the survey for a mixture of points, vouchers, or monetary reward as was standard practice for the associated market research companies. At the end of the survey, participants received a debrief explaining the aims of the study and information about who to contact if they were concerned about any medications they were taking and if they had experienced any form of discrimination in healthcare settings.

### Measures

To assess all variables of interest, we used established measures that are reliable and valid in multiple published studies.

#### Control Variables

We asked participants to report their gender, doctor’s gender, age, ethnicity, education level, employment status, if they had any diagnosed clinical conditions, how many medications they were currently taking (and which medication they will consider for this study), when in the last 6 months they were prescribed their chosen medication, and self-rated health as measured using Eriksson et al.’s single item [17].

#### Predictor

*Perceived discrimination in healthcare settings* was measured using the Discrimination in Medical Settings scale [18], consisting of seven items (Cronbach’s *α* = 0.94) asking participant to rate their experience of potential mistreatment during their GP appointment (e.g., “I was treated with less courtesy than other people”) on a five-point scale (1 = *strongly disagree*, 5 = *strongly agree*). This measure did not specify mistreatment about certain protected characteristics such as ethnicity/race to allow for a broader range of interpretation from participants and as such a wider spread of scores.

#### Potential Mediators

*The patient-doctor relationship* was measured using the Patient-Doctor Relationship Questionnaire (PDRQ-9) [19], consisting of nine items (Cronbach’s *α* = 0.94) that a person can make about their doctor (e.g., “My doctor has enough time for me”), rated on a five-point scale (1 = *not at all appropriate*, 5 = *totally appropriate*).

*Perception of doctor empathy* was measured using the Jefferson Scale of Patient’s Perceptions of Physician Empathy [20], consisting of five items (Cronbach’s *α* = 0.89) about the empathy shown by their doctor (e.g., “Understands my emotions, feelings and concerns”) rated on a five-point scale (1 = *strongly disagree*, 5 = *strongly agree).*

*Perceived quality of healthcare service* was measured using the perceived service quality subscale from the Health Service Quality scale [21]. This consists of four items (Cronbach’s *α* = 0.93) about the service provided by GP Clinics (e.g., “The overall quality of the service provided by the GP clinic is excellent”), rated on a seven-point scale (1 = *strongly disagree,* 7 = *strongly agree*).

*The expectation of side effects* was measured by adapting the side-effect attribution scale [22], asking participants to rate the extent to which they had expected to experience 50 of the most common medication side effects before starting to take their target medication (Cronbach’s *α* = 0.98) on a five-point scale (1 = *not at all expected,* 5 = *strongly expected*).

#### Dependent Variables

*Experience of side effects* was measured using the side-effect attribution scale [22], which asks participants whether they have experienced any of the following 50 symptoms (made up of 50 of the most common medication side effects) since they had been taking their medication (*yes/no*). For those that responded “*yes*,” they were then asked to rate each symptom on a five-point scale (1 = *definitely not a side-effect*, 5 = *definitely a side effect*).

*Medication adherence* was measured using the Medication Adherence Report Scale [23], which asks participants to rate the frequency of five non-adherent behaviours (Cronbach’s *α* = 0.85) (e.g. “I forget to take (name of medication)”) on a five-point scale (1 = *always,* 5 = *never*).

### Analysis

All analyses were carried out in SPSS version 26. Seventy-nine participants (9.86%) had missing data on at least one variable. None of the variables had more than 3.6% of data missing (we included answers of “don’t know” as missing data). All missing data was estimated using multiple imputation. Due to small cell counts, we had to collapse the gender categories: transgender female, transgender male, gender variant/non-conforming and not listed into an “other category” for analysis.

We examined the data for outliers using box plots, and the only clear outliers occurred in the Age variable. Two participants gave their year of birth instead of their age in years. As such, we were able to calculate an estimated age in these instances as follows: e.g., a 1996 birth year would mean the participant was 26 or 27 depending on when in the year they were born, as such their age would be calculated as 26.5. Box plots also indicated potential outliers for the variables: number of medications currently being taken, side-effect expectations total score, and side-effect experience total score; however, upon examination of the data, these could not be reasonably assumed to be “bad” data and therefore were included in the analyses.

We next examined the extent to which the variables met the assumption of normality. Only the number of medications currently being taken had a skewness and kurtosis outside the acceptable range of − 2 to + 2 and − 7 to + 7 respectively [24, 25]. This variable was *x*-transformed using the square root transformation.

Negative binomial and linear regressions were carried out to assess associations between perceived discrimination and side-effect experience and adherence whilst controlling for background characteristics that were correlated with the dependent variables. We tested mediation models based on 10,000 bootstrap samples [26] to investigate the role of the potential mediators between perceived discrimination and the dependent variables. Mediation analysis with bootstrapping uses random resampling (bootstrapping) to estimate the indirect effect of the independent variable (perceived discrimination) on the dependent variable (side-effect experience or adherence) through a mediator (e.g., patient-doctor relationship, expectation of side effects).

We also investigated the interaction between perceived discrimination and racial/ethnic groups, to see if the strength of the relationships between perceived discrimination, side-effect experience, and adherence varied between different racial/ethnic groups. We did this by comparing the relationship between perceived discrimination, side-effect experience, and adherence in BAME groups compared to White participants in the first instance, and then altered the reference group to look for any differences between BAME groups themselves.

## Results

### Participant Characteristics

**Fig. 1:**
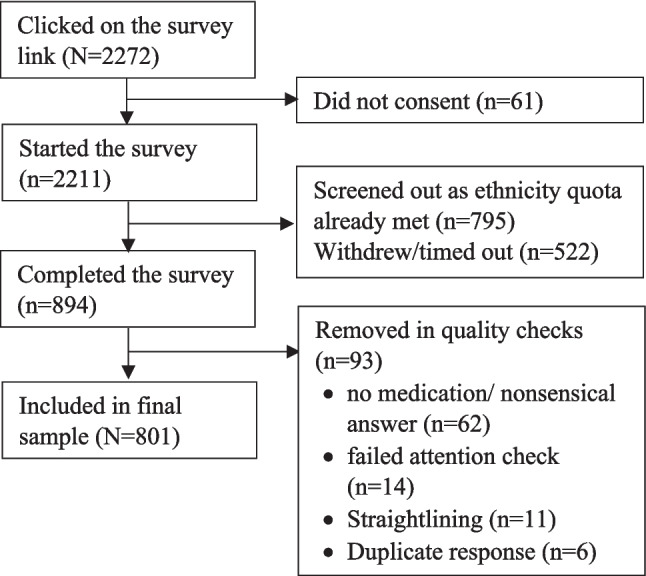
Steps taken to identify final sample of participants

The final sample contained 801 participants consisting of 280 men and 509 women, with 12 identifying as transgender male/female or non-conforming. The mean age of the sample was 34.56 years. Participant ethnicities were equally distributed across the sample: Black (24.1%), Asian (25.0%), Mixed (25.2%), and White (25.7%). The full characteristics of the sample are shown in the first two columns of Table 1. Figure 1 presents the steps involved in identifying the final sample for inclusion in the study.

**Table 1:** Participant characteristics

Variable	Total sample (*N* = 801)
Age	34.56 (12.09)
Ethnicity	
Black	193 (24.1%)
Asian	200 (25.0%)
Mixed	202 (25.2%)
White	206 (25.7%)
Gender	
Other	12 (1.5%)
Male	280 (35.0%)
Female	509 (63.5%)
Gender match with doctor	
No	268.8 (33.6%)
Yes	532.2 (66.4%)
Education	
No formal qualifications	27 (3.4%)
Below degree level	295 (36.8%)
Degree/equivalent	479 (59.8%)
Employment status	
Not working	170 (21.2%)
Working	631 (78.8%)
Self-rated health	3.29 (0.99)
Diagnosed illnesses	
Yes	348.4 (43.5%)
No	452.6 (56.5%)
No. of medications currently taking	2.29 (2.46)
When prescribed X	2.51 (1.11)

Some frequencies are not whole numbers due to the pooling of the imputed data sets

Data: mean (SD) or no. (%)

### Perceived Discrimination, Side-effect Experience, and Adherence

Across the sample, participant’s experience of perceived discrimination during their GP consultation for a medication prescribed in the last 6 months scored an average of 15.52 out of a possible 35 (SD = 7.03), with each item therefore scoring an average of 2.22. Therefore, the majority of participants reported disagreeing experiencing racial discrimination in health care [18].

The majority of participants experienced symptoms since being prescribed their selected medication (*n* = 699, 87.3%), and the average number of symptoms was 4.06 (SD = 4.80). Over half the sample (*n* = 476, 59.4%) experienced side effects while taking their medication. Side effects were identified as the symptoms that were rated as probably or definitely side effects, in the side-effect attribution scale. The average number of side effects experienced was 2.24 (SD = 3.44).

Participant’s level of adherence to their selected medication over the last 6 months was relatively high with an average of 20.31 out of 25 (SD = 4.69).

### Effect of Participant Characteristics and Perceived Discrimination on Side-effect Experience

See Table 2 for full results. In terms of participants’ demographic characteristics, there was no association with participants’ side-effect experience to their chosen medication, apart from ethnicity. Asian participants had a 22% decrease in the expected count of side effects compared to White participants.

**Table 2:** Effect of participant characteristics and perceived discrimination on side-effect experience and adherence

Variable	Number of side effects		Adherence	
	IRR (95% CI)	*P* value	B (95% CI)	*P* value
Demographics				
Age	0.99 (0.98 to 1.00)	0.074	**0.11 (0.08 to 0.13)**	** < 0.001**
Ethnicity				
Black	0.81 (0.64 to 1.03)	0.081	** − 1.97 (− 2.87 to − 1.07)**	** < 0.001**
Asian	**0.78 (0.62 to 0.98)**	**0.036**	** − 2.44 (− 3.33 to − 1.54)**	** < 0.001**
Mixed	0.83 (0.66 to 1.05)	0.124	** − 1.65 (− 2.54 to − 0.756)**	** < 0.001**
White	Reference		Reference	
Gender				
Other	1.81 (0.95 to 3.42)	0.070	− 0.21 (− 2.89 to 2.47)	0.877
Male	0.88 (0.74 to 1.05)	0.158	− 0.42 (− 1.10 to 0.27)	0.233
Female	Reference		Reference	
Gender match with doctor				
No	1.09 (0.91 to 1.30)	0.360	**1.20 (0.51 to 1.89)**	** < 0.001**
Yes	Reference		Reference	
Education				
No formal qualifications	1.02 (0.64 to 1.62)	0.931	** − 2.55 (− 4.36 to − 0.75)**	**0.006**
Below degree level	0.94 (0.79 to 1.12)	0.511	− 0.22 (− 0.90 to 0.46)	0.525
Degree/equivalent	Reference		Reference	
Employment status				
Not working	1.03 (0.84 to 1.27)	0.744	0.20 (− 0.77 to 0.81)	0.960
Working	Reference		Reference	
Self-rated health	**0.78 (0.72 to 0.84)**	** < 0.001**	** − 0.43 (− 0.75 to 0.11)**	**0.009**
Diagnosed illnesses				
Yes	**2.07 (1.75 to 2.45)**	** < 0.001**	**0.69 (0.04 to 1.35)**	**0.038**
No	Reference		Reference	
No. of medications taking	**1.10 (1.06 to 1.14)**	** < 0.001**	− 0.02 (− 0.15 to 0.12)	0.829
When prescribed X	**1.11 (1.03 to 1.20)**	**0.005**	**0.41 (0.12 to 0.70)**	**0.005**
Perceived discrimination	**1.03 (1.01 to 1.04)**^**a**^	** < 0.001**	** − 0.17 (− 0.21 to − 0.12)**^**b**^	** < 0.001**

*IRR* incidence rate ratio

^a^Controlling for ethnicity, self-rated health, Illness diagnosis, no of medications currently taking, and when they were prescribed the medication, which were significantly associated with number of side effects

^b^Controlling for age, ethnicity, gender match, education, self-rated health, illness diagnosis, and when they were prescribed the medication, which were significantly associated with adherence

With regards to health-related variables, participants with better self-rated health were less likely to experience side effects, with each additional point increase in self-rated health being associated with a 22% decrease in the expected count of side effects. Participants who had a diagnosed illness had a 107% increase in the expected count of side effects compared to those without a diagnosis. Participants who were taking more medications had a 10% increase in the expected count of side effects for every medication they were taking. Participants who had been prescribed their chosen medication for a longer period had an 11% increase in the expected count of side effects for each additional month they had been prescribed their medication.

For perceived discrimination, participants who had higher perceived discrimination scores were more likely to experience side effects, with each additional point increase in perceived discrimination being associated with a 3% increase in the expected count of side effects (see Table 2).

There was a significant interaction between participants’ ethnicity and perceived discrimination on side-effect experience. Participants who identified as Black or Mixed race were less likely to experience side effects, with each additional point increase in perceived discrimination associated with a 4% decrease in the expected count of side effects, compared to participants of White ethnicity with the same discrimination scores. See Table 3 for full results. There was no difference in the expected count of side effects for the same level of perceived discrimination between BAME groups themselves (see supplementary material).

**Table 3:** Interaction between ethnicity and perceived discrimination on side-effect experience

Variable	Number of side effects	
	Adjusted IRR^a^ (95% CI)	*P* value
Ethnicity		
Black	1.81 (0.99 to 3.28)	0.052
Asian	1.48 (0.82 to 2.67)	0.192
Mixed	1.76 (0.96 to 3.23)	0.069
White	Reference	
Self-rated health	**0.89 (0.82 to 0.98)**	**0.014**
Diagnosed illnesses		
Yes	**1.77 (1.45 to 2.15)**	** < 0.001**
No	Reference	
No. of medications currently taking	**1.04 (1.00 to 1.08)**	**0.038**
When prescribed X	**1.09 (1.01 to 1.18)**	**0.034**
Perceived discrimination	**1.05 (1.03 to 1.08)**	** < 0.001**
Black × perceived discrimination	**0.96 (0.93 to 0.99)**	**0.042**
Asian × perceived discrimination	0.97 (0.94 to 1.01)	0.106
Mixed × perceived discrimination	**0.96 (0.93 to 0.99)**	**0.035**
White × perceived discrimination	Reference	

^a^Controlling for ethnicity, self-rated health, illness diagnosis, no of medications currently taking, and when they were prescribed the medication, which were significantly associated with number of side effects

### Effect of Participant Characteristics and Perceived Discrimination on Adherence

See Table 2 for full results. In terms of participants’ demographic characteristics, there was no association between gender and employment status with participants’ self-reported adherence. However, older participants were more likely to adhere to their mediation with each additional year in age associated with a 0.11 increase in adherence score. In terms of ethnicity, participants of Black, Asian, or Mixed ethnicity were less likely to adhere to their medication with 1.97, 2.44, and 1.65 lower adherence scores (respectively) compared to White participants. Participants’ whose gender did not match that of the doctor who prescribed their chosen medication had 1.20 increase in adherence scores compared to those who’s gender did align. Participants with no formal qualifications had 2.55 lower adherence scores compared to those with degree level qualifications.

With regards to health-related variables, there was no association between the number of medications participants were taking and adherence. However, participants with higher self-rated health were less likely to adhere to their medication, with each increase in self-rated health score associated with a 0.43 decrease in adherence score. Participants with a diagnosed illness had a 0.69 increase in adherence score compared to those without a diagnosed illness. Finally, participants who were prescribed their medication longer ago were more likely to adhere to their medication with each month increase associated with a 0.41 increase in adherence score.

For perceived discrimination, participants who had higher perceived discrimination scores were less likely to adhere to their selected medication, with each additional point increase in perceived discrimination associated with a 0.17 decrease in the adherence scores.

In addition, there was no significant interaction effect between participants’ ethnicity and perceived discrimination on adherence. See Table 4 with White groups as the comparator and supplementary material for BAME groups as the comparator.

**Table 4:** Interaction between ethnicity and perceived discrimination on adherence

Variable	Adherence	
	Adjusted *B*^a^ (95% CI)	*P* value
Age	**0.07 (0.04 to 0.10)**	** < 0.001**
Ethnicity		
Black	− 0.47 (− 2.54 to 1.59)	0.653
Asian	− 1.29 (− 3.31 to 0.72)	0.208
Mixed	− 0.28 (− 2.36 to 1.79)	0.790
White	Reference	
Gender match with doctor		
No	**0.71 (0.06 to 1.36)**	**0.032**
Yes	Reference	
Education		
No formal qualifications	** − 2.15 (− 3.81 to − 0.49)**	**0.011**
Below degree level	− 0.48 (− 1.11 to 0.16)	0.141
Degree/equivalent	Reference	
Self-rated health	− 0.21 (− 0.53 to 0.11)	0.202
Diagnosed illnesses		
Yes	− 0.48 (− 1.14 to 0.18)	0.152
No	Reference	
When prescribed X	0.20 (− 0.07 to 0.47)	0.141
Perceived discrimination	** − 0.14 (− 0.23 to − 0.04)**	**0.004**
Black × perceived discrimination	− 0.05 (− 0.18 to 0.07)	0.395
Asian × perceived discrimination	− 0.03 (− 0.15 to 0.09)	0.600
Mixed × perceived discrimination	− 0.04 (− 0.17 to 0.09)	0.525
White × perceived discrimination	Reference	

^a^Controlling for age, ethnicity, gender match with doctor, education, self-rated health, illness diagnosis, and when they were prescribed the medication, which were significantly associated with adherence

### Exploring Mediators of the Relationship Between Perceived Discrimination and Side-effect Experience and Adherence

We explored the possible mediators of perceived quality of health care, patient doctor relationship, perceived doctor empathy, and side-effect expectations between perceived discrimination and side-effect experience and adherence. See the supplementary material for full mediation results.

Of these, we found the relationship between perceived discrimination and side-effect experience was fully mediated by the patient doctor relationship and side-effect expectations. Inclusion of the patient doctor relationship as a mediator removed the effect of perceived discrimination on side-effect experience from *B* = 0.033 (95% CI 0.004 to 0.04), *p* = 0.01 to *B* = 0.021 (95% CI 0.006 to 0.03), *p* = 0.102. This mediation effect was significant, *B* = 0.012 (95% CI 0.03 to 0.02), *p* = 0.006). Inclusion of side-effect expectations as a mediator removed the effect of perceived discrimination on side-effect experience from *B* = 0.048 (95% CI 0.0294 to 0.05), *p* < 0.001 to *B* = − 0.011 (95% CI − 0.04 to 0.01), *p* = 0.36. This mediation effect was significant, *B* = 0.059 (95% CI 0.0407 to 0.08), *p* < 0.001.

The relationship between perceived discrimination and adherence was only partially mediated by side-effect expectations. Inclusion of the mediation reduces the effect of perceived discrimination on adherence from *B* = − 0.2070 (95% CI − 0.2515 to −0.1624), *p* < 0.001 to *B* = − 0.1253 (95% CI − 0.1747 to − 0.0760), *p* < 0.001. This mediation effect was significant, *B* = − 0.0816 (95% CI − 0.1118 to − 0.0538), *p* < 0.05.

## Discussion

This is one of the first studies to explore the relationship between perceived discrimination in healthcare settings and patients’ experience with their medication. We found that perceived discrimination was significantly associated with increased side-effect experience, and this relationship was fully mediated by the patient-doctor relationship and side-effect expectations. This suggests that negative expectations and contextual factors of the GP consultation as a result of the perceived discrimination could be contributing to the experience of medication side effects, through the nocebo effect [27]. Not only was perceived discrimination associated with side-effect experience, but it was also significantly associated with decreased medication adherence levels. However, this was not mediated by participants’ side-effect experience, suggesting it was not the side effects causing the lowered adherence, but other factors, such as side-effect expectations, which were found to partially mediate the relationship. This highlights the importance of perceived discrimination in initial GP consultation where patients are first prescribed their medication in potentially affecting both side-effect experience and adherence.

There was a main effect of ethnicity on side-effect experience, and an interaction between ethnicity and perceived discrimination on side-effect experience; however, not in the direction one would necessarily expect. Participants who identified as Asian were less likely to report side effects than White participants. In addition, participants who identified as Mixed race were less likely to report side effects compared to White participants with the same level of perceived discrimination. There could be multiple explanations for this. First, there may be differences in how these groups attribute side effects, with White groups more willing to attribute these to the medication than other potential causes than BAME groups. Second, there may be differences in the tolerance of discrimination between ethnic groups, with BAME groups being more accepting of this behavior as it is something they unfortunately encounter more regularly as part of their lives than their White counterparts [28]. Thirdly, White groups may be overestimating the amount of racial discrimination their group experiences. For example, within workplaces in the USA, there is evidence that organizational diversity initiatives increase White Americans’ concerns about the respect and value afforded toward their racial group and increases their perceptions of anti-White bias [29]. Finally, it is possible that the White group’s experiences of discrimination could be linked to the perceived threat from the increased percentage of medical staff representing BAME groups in the UK [30, 31].

There was a main effect of ethnicity on adherence, with those in minority ethnic groups reporting lower adherence to their medication compared to White participants, supporting what is widely reported in the adherence literature [32]. There was no interaction between ethnicity and perceived discrimination on adherence. Taken together, these findings suggest it is not due to increased side-effect experience or perceived discrimination that BAME groups have lower adherence but instead other factors at the point at which BAME patients are prescribed medication such as their medication beliefs, use of complementary and alternative medicines, and language barriers that warrant attention [32].

### Limitations

There are however limitations of the study that we need to consider. The first is the cross-sectional nature of the study. As such, we cannot establish the direction of the relationships identified, and therefore the results should be interpreted in caution and in the exploratory nature for which they are intended. Second, the perceived discrimination measure we used was open to interpretation and did not specify certain protected characteristics such as ethnicity/race. This was to allow for a broader range of interpretation from participants and as such a wider spread of scores. However, it is possible that limiting this to perceived discrimination due to ethnicity/race may have altered the interactions with ethnicity. Thirdly, we recruited an online sample who were particularly well-educated (59.8% had degree level education). While the validity of data can be questioned due to concerns that participants do not read the questions properly or that they may be distracted with other tasks [33], this may not be an issue [34], and was offset by our exclusion of participants who failed the attention checks. Selection bias is more problematic, however, as how well market research panels are representative of the general population who are prescribed medications is uncertain. Finally, participants could choose any medication prescribed in the last 6 months and had to think back to the GP appointment when they were prescribed it. As such, this could be open to biases in terms of recall, and participants may have been more inclined to choose a medication which they felt particularly strongly (positively or negatively) about (if they had been prescribed more than one in the past 6 months). For example, we may see stronger relationships for medication that has a more negative reputation, e.g., statins [35]. Finally, another potential limitation concerns the fact that we did not include an ethnicity concordance variable with the participants’ doctor. This was because it may be hard for participants to correctly identify their doctor’s ethnicity. However, given the focus of this study on discrimination, the absence of ethnicity concordance information likely produces unmeasured confounding.

### Implications and Future Research

This study has helped to identify perceived discrimination in healthcare settings as a potential nocebo-related predictor which is modifiable and could serve as a critical intervention point to reduce patient’s side-effect experience and improve adherence to their medications. The results suggest that perceived discrimination seems to affect the nocebo response through increased side-effect expectations. Several strategies have shown promise in directly addressing negative expectations to reduce the nocebo effect, such as information framing, reducing the impact of negative media coverage, and educating people about the nocebo effect [36]. However, this study suggests indirect causes of negative expectations such as perceived discrimination also need to be addressed across healthcare settings to improve patients’ experience with their medications across all ethnicities.

Before this can happen, future longitudinal studies are needed to confirm the identified causal relationships and to explore the types of perceived discrimination of White and BAME individuals that are experiencing in health care and how this makes patients feel to better inform potential interventions to reduce nocebo effects. This will ultimately lead to the development and testing of interventions that could be applied to help improve patient experience and general health equity, by informing the training of healthcare professionals and the development of healthcare organization policies to reduce discrimination.

## Conclusion

While perceived discrimination in healthcare settings and its effect on health outcomes has been studied previously, understanding its relationship with the nocebo effect is a current gap in this emerging field. We found that perceived discrimination in healthcare settings was significantly associated with medication side-effect experience and adherence and could be mediated through side-effect expectations. Future research is needed to confirm the identified associations through a longitudinal design, potentially leading to development of an evidence-based intervention to reduce discrimination in healthcare settings and subsequent nocebo effects while improving health equity.

## Supplementary Information

Below is the link to the electronic supplementary material.

Supplementary file1 (DOCX 29 KB)

Supplementary file2 (DOCX 79 KB)

## Data Availability

A copy of the anonymous data set can be found here: https://osf.io/qyj6k/.
