# Supplementary material for: Perceived Discrimination in Healthcare Settings is Associated with Medication Side Effects and Adherence: A Cross-Sectional Survey Representing the Four Largest Ethnic Groups in the UK
Source: J Racial Ethn Health Disparities. 2025 Mar 26;13(3):2110–9. doi: 10.1007/s40615-025-02403-y (PMC13157394; doi:10.1007/s40615-025-02403-y)
Supplement: Supplementary file 1 — Supplementary file1 (DOCX 29 KB) [file 40615_2025_2403_MOESM1_ESM.docx]

**Supplementary material 1: Interaction analyses**

Exploring interaction analyses between ethnicity and perceived discrimination on side-effect experience and adherence, whilst altering the ethnicity reference category

Table 1. Interaction between ethnicity and perceived discrimination on side-effect experience with Black ethnicity as reference group

| Variable | Number of side-effects | |
| --- | --- | --- |
|  | Adjusted IRR^a^ (95% CI) | P value |
| Ethnicity |  |  |
| White | 0.55 (0.31 to 1.01) | .052 |
| Mixed | 0.98 (0.53 to 1.80) | .935 |
| Asian | 0.82 (0.45 to 1.49) | .514 |
| Black | Reference |  |
| Self-rated health | **0.89 (0.82 to 0.98)** | **.014** |
| Diagnosed illnesses |  |  |
| Yes | **1.77 (1.45 to 2.15)** | **<.001** |
| No | Reference |  |
| No. of medications currently taking | **1.04 (1.00 to 1.08)** | **.038** |
| When prescribed X | **1.09 (1.01 to 1.18)** | **.034** |
| Perceived Discrimination | 1.01 (0.99 to 1.04) | .264 |
| White*Perceived Discrimination | **1.04 (0.99 to 1.07)** | **.042** |
| Mixed*Perceived Discrimination | 1.00 (0.96 to 1.03) | .882 |
| Asian*Perceived Discrimination | 1.01 (0.98 to 1.04) | .616 |
| Black*Perceived Discrimination | Reference |  |

Note: ^a^Controlling for Ethnicity, Self-rated health, Illness diagnosis, no of medications currently taking and when they were prescribed the medication, which were significantly associated with number of side-effects.

Table 2. Interaction between ethnicity and perceived discrimination on side-effect experience with Asian ethnicity as reference group

| Variable | Number of side-effects | |
| --- | --- | --- |
|  | Adjusted IRR^a^ (95% CI) | P value |
| Ethnicity |  |  |
| Mixed | 1.19 (0.65 to 2.18) | .578 |
| White | 0.68 (0.37 to 1.22) | .192 |
| Black | 1.22 (0.67 to 2.21) | .514 |
| Asian | Reference |  |
| Self-rated health | **0.89 (0.82 to 0.98)** | **.014** |
| Diagnosed illnesses |  |  |
| Yes | **1.77 (1.45 to 2.15)** | **<.001** |
| No | Reference |  |
| No. of medications currently taking | **1.04 (1.00 to 1.08)** | **.038** |
| When prescribed X | **1.09 (1.01 to 1.18)** | **.034** |
| Perceived Discrimination | 1.02 (0.99 to 1.05) | .054 |
| Mixed*Perceived Discrimination | 0.99 (0.96 to 1.02) | .528 |
| White*Perceived Discrimination | 1.02 (0.99 to 1.06) | .106 |
| Black*Perceived Discrimination | 0.99 (0.96 to 1.03) | .616 |
| Asian*Perceived Discrimination | Reference |  |

Note: ^a^Controlling for Ethnicity, Self-rated health, Illness diagnosis, no of medications currently taking and when they were prescribed the medication, which were significantly associated with number of side-effects.

Table 3. Interaction between ethnicity and perceived discrimination on side-effect experience with Mixed ethnicity as reference group

| Variable | Number of side-effects | |
| --- | --- | --- |
|  | Adjusted IRR^a^ (95% CI) | P value |
| Ethnicity |  |  |
| Asian | 0.84 (0.46 to 1.54) | .578 |
| Black | 1.03 (0.56 to 1.89) | .935 |
| White | 0.57 (0.31 to 1.04) | .069 |
| Mixed | Reference |  |
| Self-rated health | **0.89 (0.82 to 0.98)** | **.014** |
| Diagnosed illnesses |  |  |
| Yes | **1.77 (1.45 to 2.15)** | **<.001** |
| No | Reference |  |
| No. of medications currently taking | **1.04 (1.00 to 1.08)** | **.038** |
| When prescribed X | **1.09 (1.01 to 1.18)** | **.034** |
| Perceived Discrimination | 1.01 (0.99 to 1.04) | .403 |
| Asian*Perceived Discrimination | 1.01 (0.98 to 1.05) | .528 |
| Black*Perceived Discrimination | 1.00 (0.97 to 1.04) | .882 |
| White*Perceived Discrimination | **1.04 (1.01 to 1.08)** | **.035** |
| Mixed*Perceived Discrimination | Reference |  |

Note: ^a^Controlling for Ethnicity, Self-rated health, Illness diagnosis, no of medications currently taking and when they were prescribed the medication, which were significantly associated with number of side-effects.

| Variable | Adherence | |
| --- | --- | --- |
|  | Adjusted B^a^(95% CI) | P value |
| Age | **0.07 (0.04 to 0.10)** | **<.001** |
| Ethnicity |  |  |
| White | 0.47 (-1.59 to 2.54) | .653 |
| Mixed | 0.19 (-1.92 to 2.30) | .858 |
| Asian | -0.81 (-2.85 to 1.22) | .431 |
| Black | Reference |  |
| Gender match with doctor |  |  |
| No | **0.71 (0.06 to 1.36)** | **.032** |
| Yes | Reference |  |
| Education |  |  |
| No formal qualifications | **-2.15 (-3.81 to -0.49)** | **.011** |
| Below degree level | -0.48 (-1.11 to 0.16) | .141 |
| Degree/equivalent | Reference |  |
| Self-rated health | -0.21 (-0.53 to 0.11) | .202 |
| Diagnosed illnesses |  |  |
| Yes | -0.48 (-1.14 to 0.18) | .152 |
| No | Reference |  |
| When prescribed X | 0.20 (-0.07 to 0.47) | .141 |
| Perceived Discrimination | **-0.19 (-0.27 to -0.10)** | **<.001** |
| White*Perceived Discrimination | 0.05 (-0.07 to 0.18) | .395 |
| Mixed*Perceived Discrimination | 0.01 (-0.11 to 0.14) | .842 |
| Asian*Perceived Discrimination | 0.02 (-0.09 to 0.14) | .713 |
| Black*Perceived Discrimination | Reference |  |

Table 4. Interaction between ethnicity and perceived discrimination on adherence with Black ethnicity as the reference group

Note: ^a^Controlling for Age, Ethnicity, Gender match with doctor, Education, Self-rated health, Illness diagnosis, and when they were prescribed the medication, which were significantly associated with adherence

Table 5. Interaction between ethnicity and perceived discrimination on adherence with Asian ethnicity as the reference group

| Variable | Adherence | |
| --- | --- | --- |
|  | Adjusted B^a^(95% CI) | P value |
| Age | **0.07 (0.04 to 0.10)** | **<.001** |
| Ethnicity |  |  |
| Mixed | 1.01 (-1.05 to 3.07) | .336 |
| White | 1.29 (-0.72 to 3.31) | .208 |
| Black | 0.82 (-1.22 to 2.85) | .431 |
| Asian | Reference |  |
| Gender match with doctor |  |  |
| No | **0.71 (0.06 to 1.36)** | **.032** |
| Yes | Reference |  |
| Education |  |  |
| No formal qualifications | **-2.15 (-3.81 to -0.49)** | **.011** |
| Below degree level | -0.48 (-1.11 to 0.16) | .141 |
| Degree/equivalent | Reference |  |
| Self-rated health | -0.21 (-0.53 to 0.11) | .202 |
| Diagnosed illnesses |  |  |
| Yes | -0.48 (-1.14 to 0.18) | .152 |
| No | Reference |  |
| When prescribed X | 0.20 (-0.07 to 0.47) | .141 |
| Perceived Discrimination | **-0.17 (-0.25 to -0.09)** | **<.001** |
| Mixed*Perceived Discrimination | -0.01 (-0.13 to 0.11) | .880 |
| White*Perceived Discrimination | 0.03 (-0.09 to 0.15) | .600 |
| Black*Perceived Discrimination | -0.02 (-0.14 to 0.09) | .713 |
| Asian*Perceived Discrimination | Reference |  |

Note: ^a^Controlling for Age, Ethnicity, Gender match with doctor, Education, Self-rated health, Illness diagnosis, and when they were prescribed the medication, which were significantly associated with adherence

Table 6.Interaction between ethnicity and perceived discrimination on adherence with Mixed ethnicity as the reference group

| Variable | Adherence | |
| --- | --- | --- |
|  | Adjusted B^a^(95% CI) | P value |
| Age | **0.07 (0.04 to 0.10)** | **<.001** |
| Ethnicity |  |  |
| Asian | -1.01 (-3.07 to 1.05) | .336 |
| Black | -0.19 (-2.30 to 1.92) | .858 |
| White | 0.28 (-1.79 to 2.36) | .790 |
| Mixed | Reference |  |
| Gender match with doctor |  |  |
| No | **0.71 (0.06 to 1.36)** | **.032** |
| Yes | Reference |  |
| Education |  |  |
| No formal qualifications | **-2.15 (-3.81 to -0.49)** | **.011** |
| Below degree level | -0.48 (-1.11 to 0.16) | .141 |
| Degree/equivalent | Reference |  |
| Self-rated health | -0.21 (-0.53 to 0.11) | .202 |
| Diagnosed illnesses |  |  |
| Yes | -0.48 (-1.14 to 0.18) | .152 |
| No | Reference |  |
| When prescribed X | 0.20 (-0.07 to 0.47) | .141 |
| Perceived Discrimination | **-0.18 (-0.27 to -0.09)** | **<.001** |
| Asian*Perceived Discrimination | 0.01 (-0.11 to 0.13) | .880 |
| Black*Perceived Discrimination | -0.01 (-0.14 to 0.11) | .842 |
| White*Perceived Discrimination | 0.04 (-0.09 to 0.17) | .525 |
| Mixed*Perceived Discrimination | Reference |  |

Note: ^a^Controlling for Age, Ethnicity, Gender match with doctor, Education, Self-rated health, Illness diagnosis, and when they were prescribed the medication, which were significantly associated with adherence
