# Supplementary material for: Perceived Discrimination in Healthcare Settings is Associated with Medication Side Effects and Adherence: A Cross-Sectional Survey Representing the Four Largest Ethnic Groups in the UK
Source: J Racial Ethn Health Disparities. 2025 Mar 26;13(3):2110–9. doi: 10.1007/s40615-025-02403-y (PMC13157394; doi:10.1007/s40615-025-02403-y)
Supplement: Supplementary file 2 — Supplementary file2 (DOCX 79 KB) [file 40615_2025_2403_MOESM2_ESM.docx]

**Supplementary material 2: Mediation analyses**

*Exploring mediators of the relationship between perceived discrimination and number of side-effects experienced*

a) Perceived quality of healthcare

Perceived discrimination

Perceived quality of health care

Side-effect experience

B=0.006 (95% CI -0.001 to 0.02), p=.140

B=-0.013, p=0.14

B=-0.279, p<.001

B=0.034 (95% CI 0.006 to 0.04), p=.006 (B=0.027 (95% CI 0.00007 to 0.04), p=.026)

b) Patient doctor relationship

Perceived discrimination

Side-effect experience

Patient doctor relationship

B=0.012, (95% CI 0.003 to 0.02), p=.006

B=-0.017, p=.007

B=-0.44, p<.001

B=0.033 (95% CI 0.004 to 0.04), p=.01 (B=0.021 (95% CI 0.006 to 0.03), p=.102)

c) Perceived doctor empathy

Perceived discrimination

Perceived doctor empathy

Side-effect experience

B=0.001 (95% CI -0.005 to 0.01), p=.636

B=-0.005, p=.671

B=-0.166, p<.001

B=0.034 (95% CI 0.008 to 0.04), p=.004 (B=0.032 (95%% CI 0.007 to 0.04), p=.012)

d) Side-effect expectations

Perceived discrimination

Side effect expectations

Side-effect experience

B=0.059 (95%CI 0.0407 to 0.08), p<.001

B=0.048 (95% CI 0.0294 to 0.05), p<.001 (B=-0.011 (95% CI -0.04 to 0.01), p=.36)

B=0.021, p<.001

B=1.90, p<.001

*Exploring mediators of the relationship between perceived discrimination and adherence*

a) Side effect experience

Perceived discrimination

Adherence

B = .0460, p=.008

Side effect experience

B = .0927,p=.0432

B = .0043, (95% CI .0002 to .0107), p<.05

B=-.2111, (95% CI -.2551 to -.1671), p<.001, (B = -.2153, (95% CI -.2594 to -.1712), p<.001)

b) Perceived quality of healthcare

B = .0064, (95% CI -.0094 to .0240), p>.05

Perceived discrimination

B = -.2787, p<.001

Perceived quality of health care

B = -.0229, p=.3981

Adherence

B=-.2110, (95% CI -.2552 to -.1667), p<.001, (B = -.2174, (95% CI -.2640 to -.1707), p<.001)

c) Patient doctor relationship

B = .0249, (95% CI .0072 to .0458), p<.05

B = -.4433, p<.001

Perceived discrimination

Patient doctor relationship

Adherence

B = -.0562, p=.0027

B= -.2074, (95% CI -.2514 to -.1633), p<.001, (B = -.2323, (95% CI -.2790 to -.3489), p<.001)

d) Perceived doctor empathy

B = .0108, (95% CI -.0015 to .0263), p>.05

Perceived discrimination

B = -.1658, p<.001

Perceived doctor empathy

Adherence

B = -.0652, p=.0748

B= -.2102, (95% CI -.2545 to -.1660), p<.001, (B = -.2211, (95% CI -.2668 to -.1753), p<.001)

e) Side-effect expectations

B = -.0816, (95% CI -.1118 to -.0538), p<.05

Perceived discrimination

B = 1.9003, p<.001

Side effect expectations

Adherence

B = -.0430, p<.001

B= -.2070, (95% CI -.2515 to -.1624),p<.001 (B = -.1253, (95% CI -.1747 to -.0760), p<.001)
